# Identification of *Corynebacterium ulcerans* and *Erysipelothrix* sp. in Malayan pangolins—a potential threat to public health?

**DOI:** 10.1128/msphere.00551-24

**Published:** 2024-09-30

**Authors:** Hai Wang, Xiao Wang, Yilin Cao, Yiting Chen, Zanjian Zou, Xingbang Lu, Fen Shan, Jieying Tu, Jianhua Liu, Jiameng Liu, Jiaqi Sa, Niu Zhou, Shi-Ming Peng, Jie-Jian Zou, Xuejuan Shen, Junqiong Zhai, Zujin Chen, Edward C. Holmes, Wu Chen, Yongyi Shen

**Affiliations:** 1 State Key Laboratory for Animal Disease Control and Prevention, Guangdong Laboratory for Lingnan Modern Agriculture, Center for Emerging and Zoonotic Diseases, College of Veterinary Medicine, South China Agricultural University, Guangzhou, China; 2 Guangzhou Zoo, Guangzhou Wildlife Research Center, Guangzhou, China; 3 College of Veterinary Medicine, National Risk Assessment Laboratory for Antimicrobial Resistant of Microorganisms in Animals, Guangdong Provincial Key Laboratory of Veterinary Pharmaceutics Development and Safety Evaluation, Key Laboratory of Zoonosis of Ministry of Agricultural and Rural Affairs, Center for Emerging and Zoonotic Diseases, South China Agricultural University, Guangzhou, China; 4 Guangdong Provincial Wildlife Monitoring and Rescue Center, Guangzhou, China; 5 School of Medical Sciences, University of Sydney, Sydney, New South Wales, Australia; 6 Guangdong Provincial Key Laboratory of Zoonosis Prevention and Control, Guangzhou, Guangdong, China; Nanjing University of Chinese Medicine, Nanjing, Jiangsu, China

**Keywords:** pangolin, *Corynebacterium ulcerans*, *Erysipelothrix* sp., zoonosis

## Abstract

The discovery of severe acute respiratory syndrome-coronavirus-2-like and Middle East respiratory syndrome-coronavirus-like viruses in Malayan pangolins has raised concerns about their potential role in the spread of zoonotic diseases. Herein, we describe the isolation and whole-genome sequencing of potentially zoonotic two bacterial pathogens from diseased Malaysian pangolins (*Manis javanica*)—*Corynebacterium ulcerans* and *Erysipelothrix* sp. The newly identified species were designated as *C. ulcerans* P69 and *Erysipelothrix* sp. P66. *C. ulcerans* P69 exhibited 99.2% whole-genome nucleotide identity to human bacterial isolate 4940, suggesting that it might have zoonotic potential. Notably, *C. ulcerans* P69 lacked the diphtheria toxin (*tox*) gene that is widely used in vaccines to protect humans from corynebacterial infection, which suggests that the current vaccine may be of limited efficacy against this pangolin strain. *C. ulcerans* P69 also contains other known virulence-associated genes such as *pld* and exhibits resistance to several antibiotics (erythromycin, clindamycin, penicillin G, gentamicin, tetracycline), which may affect its effective control. *Erysipelothrix* sp. P66 was closely related to *Erysipelothrix* sp. strain 2-related strains, exhibiting 98.8% whole-genome nucleotide identity. This bacterium is lethal in mice, and two commercial vaccines failed to protect its challenge, such that it could potentially pose a threat to the swine industry. Overall, this study highlights that, in addition to viruses, pangolins harbor bacteria that may pose a potential threat to humans and domestic animals, and which merit attention.

**IMPORTANCE:**

This study firstly reports the presence of two potentially zoonotic bacteria, *Corynebacterium ulcerans* and *Erysipelothrix* sp., in diseased Malaysian pangolins collected in 2019. The pangolin *C. ulcerans* is lethal in mice and resists many antibiotics. It clustered with a lethal human strain but lacked the diphtheria toxin gene. Diphtheria toxin is widely used as a vaccine around the world to protect humans from the infection of corynebacteria. The lack of the *tox* gene suggests that the current vaccine may be of limited efficacy against this pangolin strain. The pangolin *Erysipelothrix* sp. is the sister clade of *Erysipelothrix rhusiopathiae*. It is lethal in mice, and two commercial vaccines failed to protect the mice against challenge with the pangolin *Erysipelothrix* sp., such that this strain could potentially pose a threat to the swine industry. These findings emphasize the potential threat of pangolin bacteria.

## INTRODUCTION

All eight species of pangolins are classified as endangered by the International Union for Conservation of Nature (IUCN). As the world’s most commonly trafficked mammal, pangolins are prone to contact with other wildlife and hence are vulnerable to pathogens that can lead to illness and death (1). Despite this, little is known about the microbial pathogens that infect these species. Following the emergence of COVID-19 it was discovered that pangolins can be infected by both severe acute respiratory syndrome-coronavirus-2-related and Middle East respiratory syndrome-coronavirus-related viruses (2–4). Further investigations revealed the presence of other potential zoonotic viruses, such as human parainfluenza 3, human respiratory syncytial viruses, and murine respirovirus, in these animals (5, 6). Bacterial infections also pose a significant threat to both human and animal health, particularly at the wildlife-domestic and animal-human interfaces, where certain bacteria can transmit and cause serious disease (7). Previous studies indicated that the main pathogenic bacteria in pangolins are *Escherichia coli, Klebsiella pneumoniae, Proteus vulgaris, Streptococcus faecalis*, and *Staphylococcus* sp. (8). Although bacteria pose a clear threat to the health of these endangered animals, our understanding of the bacterial pathogens that infect pangolins is limited.

Corynebacteria are a group of bacteria that can infect a range of domestic and wild animals, including dogs, cats, cows, horses, and squirrels, and in some cases, animals serve as the source of corynebacteria that subsequently cause disease in humans (9). The most notable species in this group is *Corynebacterium diphtheria*, which causes diphtheria in humans. Despite extensive vaccine usage, diphtheria is yet to be eradicated. *Corynebacterium ulcerans* is also known to cause diphtheria and diphtheria-like infections in humans, and is difficult to distinguish from diphtheria in clinical practice. Transmission of *C. ulcerans* among wildlife, domestic animals, and humans is commonplace (10). *Erysipelothrix* is a common commensal or a pathogen of wild and domestic mammals, birds, and fish (11). The best-known species in this genus is *Erysipelothrix rhusiopathiae* which is the cause of erysipelas in pigs, with sheep, rabbits, cattle, turkeys, and rats also subject to infection (12). *Erysipelothrix* is not only the primary causative agent of swine erysipelas but also has the ability to cause erysipeloid in humans (13). Human infection due to *E. rhusiopathiae* is occupationally related, principally occurring as a result of contact with contaminated animals, their products, or waste (14).

Previous reports have primarily focused on parasitic and viral infections in pangolins (2–4, 15, 16), leaving a noticeable gap in our understanding of their bacterial infections. Herein, we detected and isolated *Corynebacterium ulcerans* and *Erysipelothrix* sp. from pangolins for the first time. Phylogenetic analysis based on the core genes was performed, and the virulence of these bacteria was assessed through mice challenge experiments. The antimicrobial susceptibility of the two strains was also tested.

## RESULTS

### Bacterial species identification and genome characteristics

Bacterial colonies isolated from diseased Malaysian pangolins P66 and P69 were subjected to Gram staining and microscopic examination (Fig. S1). Their 16S rRNA gene was amplified and sequenced for bacteriological species identification. Two bacteria, designed as *Corynebacterium* sp. P69 and *Erysipelothrix* sp. P66, were subsequently identified. The 16S rRNA gene sequence of *Corynebacterium* sp. P69 exhibited more than 99% identity to the corresponding sequences of *C. ulcerans* NCTC 7910 (NR_029292.1, 99.46% identity) and *C. pseudotuberculosis* CD070 (NR_117210.1, 99.52% identity). The 16S rRNA gene sequence of *Erysipelothrix* sp. P66 exhibited >99% identity to *E. rhusiopathiae* ATCC 19414 (NR_040837.1, 100% identity), *E. tonsillarum* DSM 14972 (NR_113036.1, 99.80% identity), and *E. piscisicarius* 15TAL0474 (NR_170394.1, 99.61% identity).

**Fig 1:**
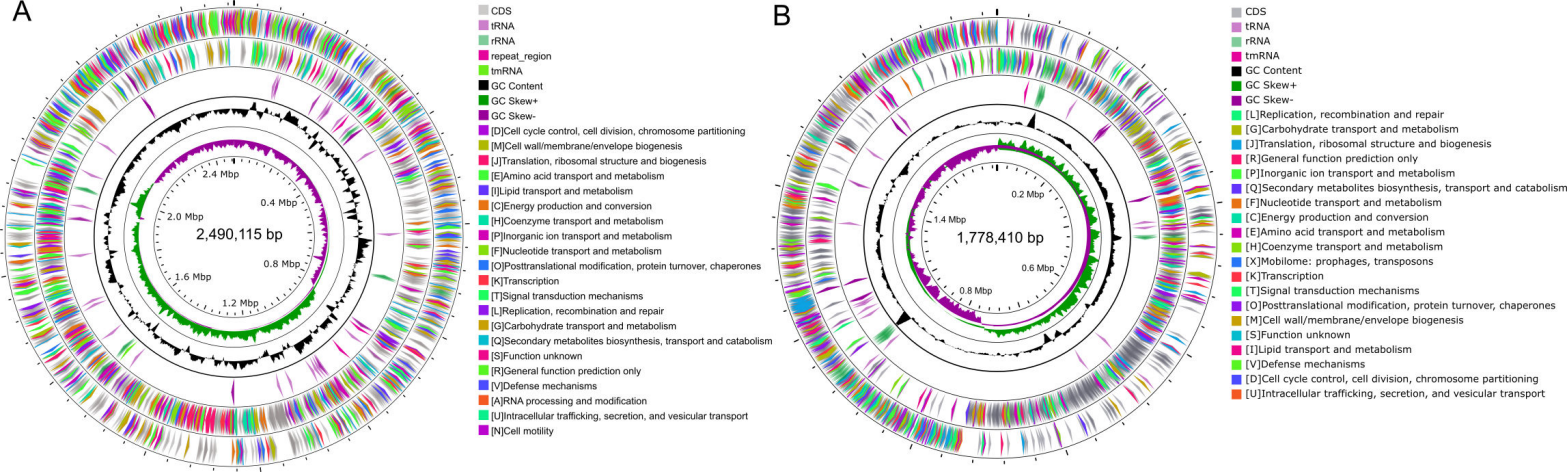
Circular genome map of *C. ulcerans* P69 and *Erysipelothrix* sp. P66. (**A**) *C. ulcerans* P69 and (**B**) *Erysipelothrix* sp. P66. Circles are shown from the outside to the inner. Ring 1, CDSs on forward strand (colored by COG categories). Ring 2, CDSs on reverse strand (colored by COG categories). Ring 3, RNA genes (tRNAs, rRNA, tmRAN, and repeat region). Ring 4, GC content. Ring 5, GC skew.

**Fig 2:**
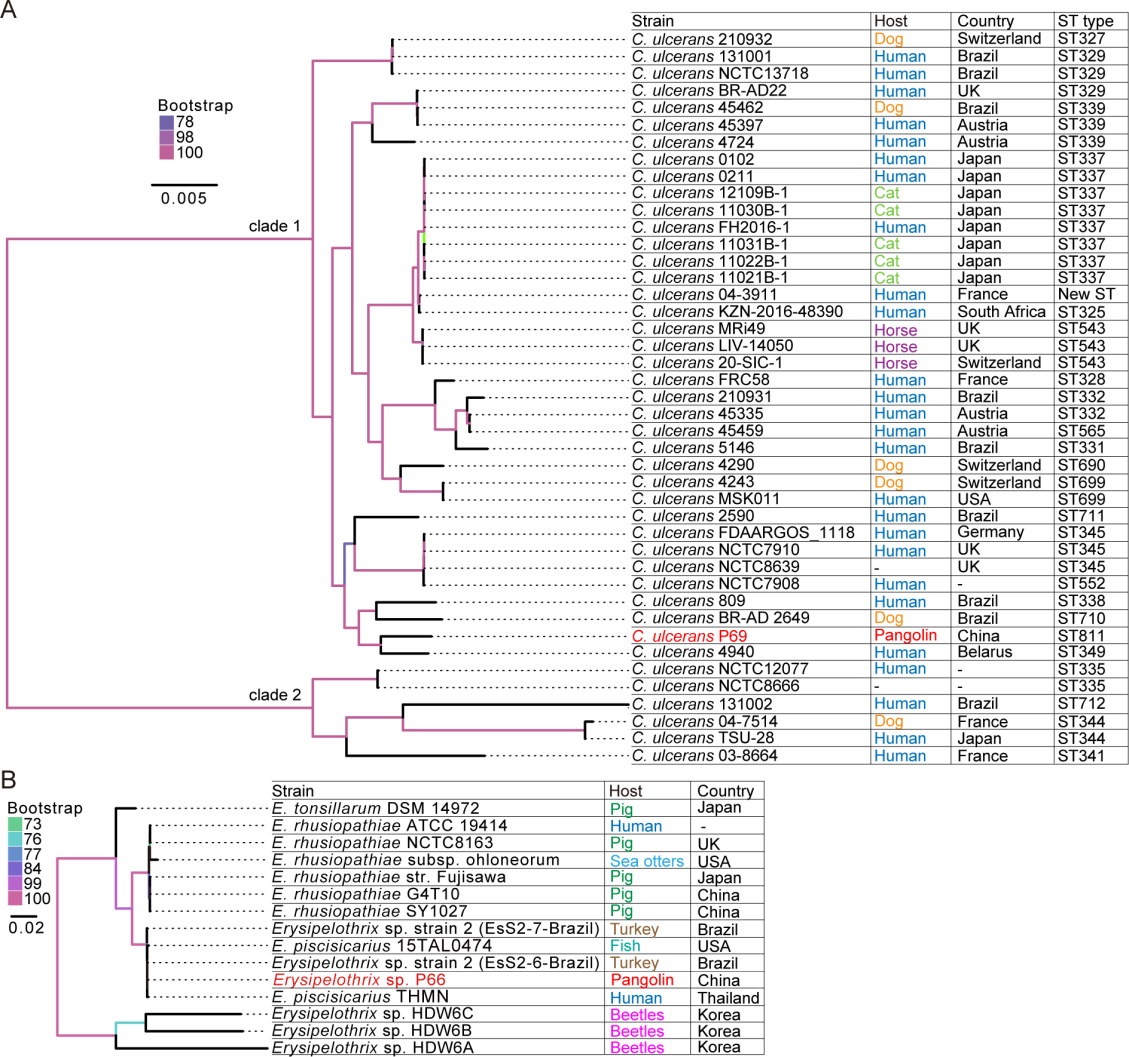
Phylogenetic trees of *Corynebacterium ulcerans* and *Erysipelothrix* sp. (**A**) *Corynebacterium ulcerans* and (**B**) *Erysipelothrix* sp. Maximum likelihood (ML) trees were estimated based on 1,601 and 10 core genes for 43 strains of *Corynebacterium ulcerans* and 15 strains of *Erysipelothrix* sp., respectively. The ML trees were rooted on the mid-point of the longest branch. Information of the host, country, and sequence type (ST) of the bacteria is shown on the right of the trees.

The genomes of these bacterial species were sequenced by combining Oxford Nanopore platform and Illumina NovaSeq PE150 read data. The complete genome of *Corynebacterium* sp. P69 contains a circular chromosome of 2,490,115 bp with 53.3% GC content. A total of 2,291 protein-coding sequences (CDSs), 51 tRNA, and 12 rRNA genes were predicted (Fig. 1A). A majority of the CDSs (1,907/2,291, 83.24%) could be assigned a putative function according to 18 Clusters of Orthologous Group (COG) categories (Fig. 1A). The complete genome of *Erysipelothrix* sp. P66 contains a circular chromosome of 1,778,410 bp with 37.3% GC content (Fig. 1B). A total of 1,766 CDSs, 55 tRNA, and 18 rRNA genes were predicted (Fig. 1B). A majority of the CDSs (1466/1766, 83.01%) could be assigned a putative function according to 20 COG categories (Fig. 1B). Whole-genome average nucleotide identity (ANI) and core genome phylogenetic analysis confirmed that *Corynebacterium* sp. P69 belongs to *C. ulcerans* while *Erysipelothrix* sp. P66 belongs to *Erysipelothrix* sp. strain 2-related isolates (Fig. 2; Fig. S2). As a consequence, *Corynebacterium* sp. P69 was further designed as *C. ulcerans* P69.

### Comparative genomic analysis

Unlike the other 42 strains, *C. ulcerans* P69 belongs to sequence type (ST) 811 (Fig. 2A). A phylogenetic tree estimated from 1,601 core genes revealed that *C. ulcerans* strains form two major clades (Fig. 2A). *C. ulcerans* P69 belongs to clade 1 and was most closely related to human isolate 4940 from Belarus, and forming a sister clade with human isolate 809 and canine isolate BR-AD 2649 (Fig. 2A). Analysis of the ANI supported the existence of the two clades revealed by phylogenetic analysis: the ANI within each clade was high, from 97.65% to 99.91% in clade 1 and 98.40% to 99.99% in clade 2 with a distance of 95.67%–96.45% between clade 1 and clade 2 (Fig. S2A). *C. ulcerans* P69 shared the highest identity (99.2%) with human isolate 4940 (Fig. S2A).

The pan-genome analysis of 43 *C*. *ulcerans* strains identified 1,601 core genes (Table S2). The top five functional categories according to COG classification were as follows: function unknown (S; 282/1,601, 17.6%), translation, ribosomal structure, and biogenesis (J; 137/1,601, 8.6%), amino acid transport and metabolism (E; 123/1,601, 7.7%), inorganic ion transport and metabolism (P; 111/1,601, 6.9%), and transcription (K; 93/1,601, 5.8%) (Fig. S3A). *C. ulcerans* P69 and *C. ulcerans* 4940 had 168 and 115 unique protein clusters, respectively, most of which were unannotated or unknown functions. Of these, 21.4% (36/168) and 12.2% (14/115) were prophage genes (Fig. S3B and C).

*Erysipelothrix* sp. P66 clustered with two *Erysipelothrix* sp. strain 2 bacteria isolated from turkeys and two *E. piscisicarius* strains isolated from fish and humans (Fig. 2B). *Erysipelothrix* sp. P66 had a whole-genome identity of ~98.8% to the four strains related to *Erysipelothrix* sp. strain 2 and 88.4% to the *E. rhusiopathiae* strains (Fig. S2B). The pan-genome analysis of *Erysipelothrix* sp. P66 and the four strains related to *Erysipelothrix* sp. strain 2 identified 1,178 core genes (Table S4). The top five functional categories according to COG classification were as follows: function unknown (S; 276/1,178, 23.4%), translation, ribosomal structure, and biogenesis (J; 147/1,178, 12.5%), transcription (K; 112/1,178, 9.5%), carbohydrate transport and metabolism (G; 102/1,178, 8.7%), and replication, recombination, and repair (L; 96/1,178, 8.1%) (Fig. S4A). *Erysipelothrix* sp. P66, *E. piscisicarius* THMN, *E. piscisicarius* 15TAL0474, *Erysipelothrix* sp. strain 2 (EsS2-6-Brazil), and *Erysipelothrix* sp. strain 2 (EsS2-7-Brazil) had 137, 434, 335, 81, and 53 unique protein clusters, respectively, with the main functional categories being unannotated (N/A: 41/137, 29.9%; 63/434, 14.5%; 38/335, 11.3%; 21/81, 25.9%; 18/53, 34%), replication, recombination, and repair (L: 41/137, 29.9%; 40/434, 9.2%; 128/335, 38.2%; 7/81, 8.6%; 8/53, 15.1%), and function unknown (S: 27/137, 19.7%; 45/434, 10.4%; 44/335, 13.1%; 7/81, 8.6%; 7/53, 13.2%) (Fig. S4B).

### Analysis of virulence and antimicrobial resistance genes

**Fig 3:**
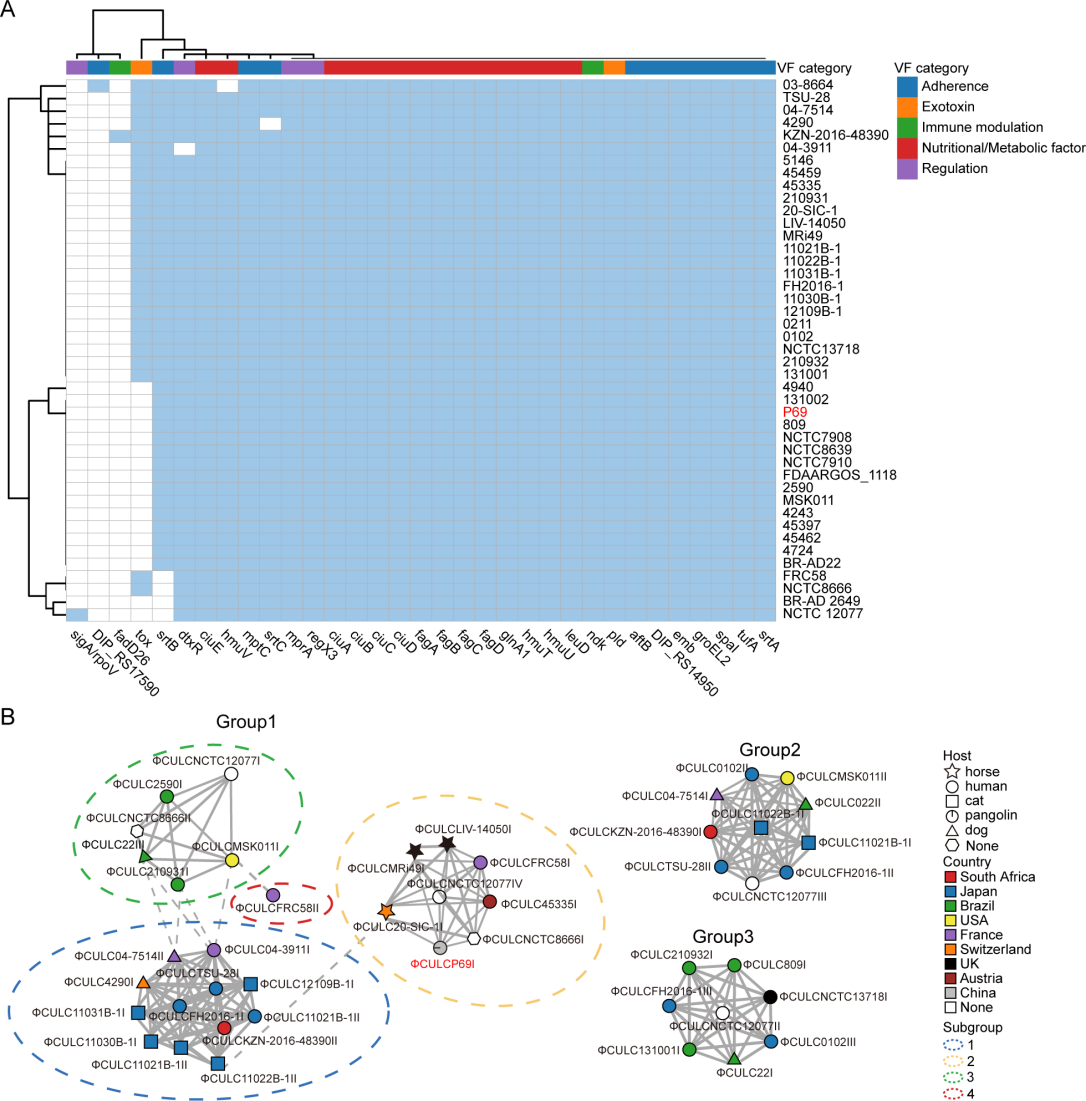
Virulence gene profiles and network of putative prophages of *C. ulcerans*. (**A**) The heatmap of virulence genes. The presence of virulence genes is displayed in blue boxes, while absence is marked in white boxes. The clustering on both the horizontal and vertical axes was performed using the ward.D2 method. (**B**) The connections between prophages of different strains were based on CDS similarity. Nodes represent individual prophages, with node shape indicating host species and node color indicating country of origin. Line thickness represents the number of similar CDSs between connected prophages with thicker lines indicating a higher number of similar CDSs. Solid gray lines represent connections to all points within the group. Dashed lines represent limited connections between points.

A total of 33 virulence genes belonging to five virulence categories were detected among the 43 *Corynebacterium ulcerans* strains analyzed in this study (Fig. 3A). In *C. ulcerans* P69, 29 virulence genes were identified, although the *tox* gene was absent (Fig. 3A). *C. ulcerans* can acquire virulence through phage lysogenization (17). A total of 45 prophages were detected in 28 *C*. *ulcerans* strains (Table S5). In *C. ulcerans* P69, a 40.3-kb prophage region, named ΦCULCP69I, was identified. Based on the pairwise comparisons of sequence identity, the 45 prophages were classified into three groups, with Group 1 further subdivided into four subgroups (Fig. 3B). ΦCULCP69I belonged to subgroup 2, showing partial genomic features and sequence similarities with prophages from *C. ulcerans* strains LIV-14050, FRC58, 45335, NCTC8666, NCTC 12077, MRi49, and 20-SIC-1 (Fig. S5B). Fifteen of these prophages carry DT-encoding *tox* genes, and three carry a putative virulence factor similar to RhuM (18) (Fig. S5A through C). ΦCULCP69I did not contain virulence-related genes.

**Fig 4:**
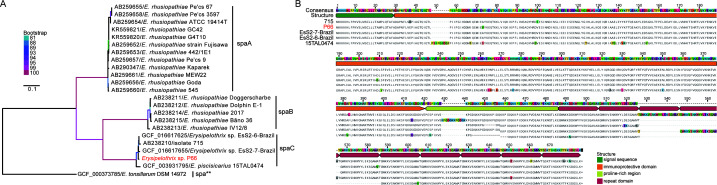
Comparative analysis of the surface protective antigen (Spa). (**A**) ML tree of *Erysipelothrix* strains based on the amino acid sequences of Spa protein. The tree was rooted with *E. tonsillarum* DSM 14972. (**B**) Animo acid sequence alignment of the *spaC* gene from five strains related to *Erysipelothrix* sp. strain 2. The different colored bars highlight the 29-amino-acid signal sequence (green), immunoprotective domain (yellow), proline-rich region (cyan), and 20-amino-acid repeat domain (red). Amino acid differences are highlighted with different colored backgrounds.

Blast analysis revealed the presence of 13 genes associated with *E. rhusiopathiae* virulence traits (*spa, nanH.1, nanH.2, cpsA,* ERH_1356*, intl-like, rspA, rspB, algI, sub, hlyA, fbpA,* and *hlyIII*) in *Erysipelothrix* sp. P66 (Table S6). A phylogenetic tree based on the spa protein sequences revealed that *Erysipelothrix* sp. P66 clusters with spaC of other strains related to *Erysipelothrix* sp. strain 2, including strains from swine, turkeys, and fish (Fig. 4A). The amino acid sequence similarity within different types of *spa* is relatively high, ranging from 86% to 100% for *spaA*, 96.7% to 99.5% for *spaB*, and 82.8% to 96.1% for *spaC* (Fig. S6). In contrast, the similarity between different spa types ranges from 52% to 63.7% (between *spaA* and *spaB*), 52.2% to 64.3% (between *spaA* and *spaC*), and 59.1% to 65.6% (between *spaB* and *spaC*), respectively (Fig. S6). The signal sequence was conserved in all *spaC* proteins of the isolates (Fig. 4B). *Erysipelothrix* sp. P66 harbors two amino acid mutations (73V; 714E) in the immunoprotective domain region and an insertion region of 12 amino acids in the proline-rich region (Fig. 4B). Furthermore, the number of repeat regions varies among different strains: *Erysipelothrix* sp. 715 has the most with 10 repeat regions, *Erysipelothrix* sp. EsS2-6-Brazil has 9 repeat regions, *Erysipelothrix* sp. P66 and *Erysipelothrix* sp. EsS2-7-Brazil both have 8 repeat regions, while *E. piscisicarius* 15TAL0474 has 7 (Fig. 4B).

No antimicrobial resistance genes were detected in either *C. ulcerans* P69 or the *Erysipelothrix* sp. P66 genomes. Subsequent antimicrobial susceptibility testing revealed the resistance of *C. ulcerans* P69 to numerous antibiotics, including erythromycin (MIC, 256 µg/mL), clindamycin (MIC, 8 µg/mL), penicillin G (MIC, 32 µg/mL), gentamicin (MIC, 64 µg/mL), and tetracycline (MIC, 256 µg/mL) (Table 1). *Erysipelothrix* sp. P66 exhibits sensitivity to most antibiotics but has very high MIC values for vancomycin (MIC, 32 µg/mL) and gentamicin (MIC, 128 µg/mL) (Table 1).

**TABLE 1:** Antimicrobial susceptibility of *C. ulcerans* P69 and *Erysipelothrix* sp. P66

Antibiotics	MIC (μg/mL)	CLSI^*a*^		
		S≤	I	R≥
*C. ulcerans* P69				
Erythromycin	256	0.5	1	2
Tilmicosin	32	−^*b*^	−	−
Vancomycin	16	2	−	−
Clindamycin	8	0.5	1–2	4
Oxacillin	32	−	−	−
Penicillin G	32	0.12	0.25–2	4
Cotrimoxazole	16	−	−	−
Gentamicin	64	4	8	16
Tetracycline	256	4	8	16
Cefoxitin	16	−	−	−
Ceftiofur	2	−	−	−
Ciprofloxacin	2	1	2	4
Florfenicol	128	−	−	−
*Erysipelothrix* sp. P66				
Erythromycin	≤0.125	0.25	0.5	1
Tilmicosin	≤0.25	−	−	−
Vancomycin	32	−	−	−
Clindamycin	≤0.125	0.25	0.5	1
Oxacillin	≤0.125	−	−	−
Penicillin G	≤0.125	0.12	−	−
Cotrimoxazole	0.125	−	−	−
Gentamicin	128	−	−	−
Tetracycline	1	−	−	−
Cefoxitin	0.25	−	−	−
Ceftiofur	≤0.125	−	−	−
Ciprofloxacin	≤0.03	1	−	−
Florfenicol	2	−	−	−

^*a*^ Breakpoint of MIC was from the CLSI (2015, M45). S, susceptible; I, intermediate; R, resistant.

^*b*^ –, data unavailable.

### Pathogenicity assessment of *C. ulcerans* P69 and *Erysipelothrix* sp. P66

**Fig 5:**
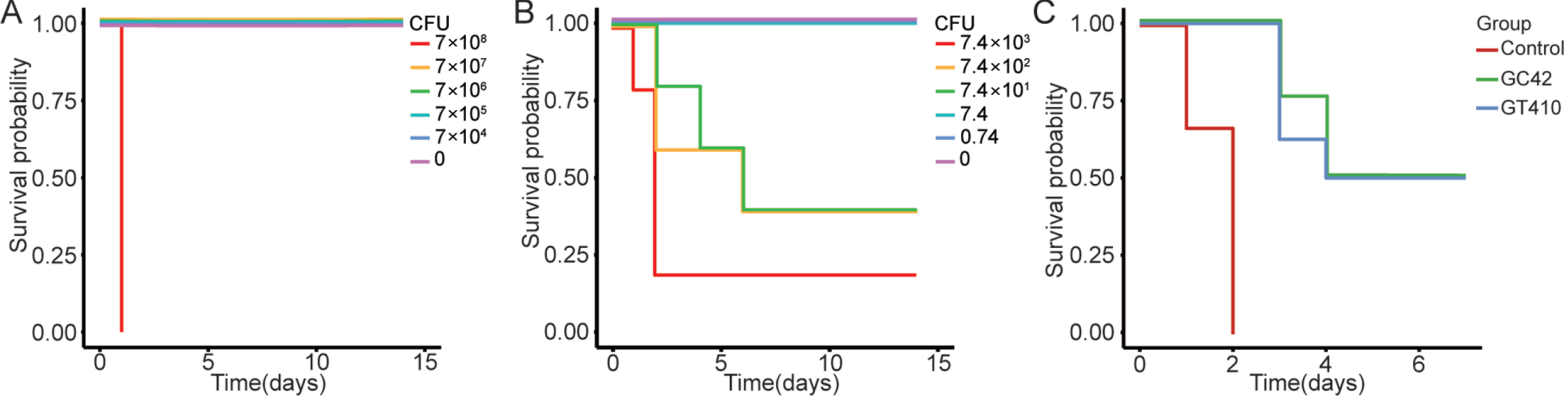
Survival curve after bacterial infection. The survival curve of mice infected with different doses of bacteria. (**A**) *C. ulcerans* P69 at 7 × 10^8^ , 7 × 10^7^, 7 × 10^6^, 7 × 10^5^, and 7 × 10^4^ CFU. *Erysipelothrix* sp. P66 (**B**) at 7.4 × 10^3^, 7.4 × 10^2^, 7.4 × 10^1^, 7.4, and 0.74 CFU. (**C**) Survival curve of two commercial vaccines’ (GC42, G4T10) protection challenge against *Erysipelothrix* sp. P66.

The ability of these bacteria to cause a lethal infection in mice was also tested (Fig. 5A and B). The 50% lethal dose (LD50) of *C. ulcerans* P69 was estimated to be 2.2 × 10^8^ CFU. Mice infected with 7 × 10^7^ CFU exhibited significant weight loss (analysis of variance and Tukey HSD test, *P* < 0.001) (Fig. S7A). Pathological changes in the liver included nodular necrosis of liver cells, eosinophilic transformation, narrowing or disappearance of liver sinuses due to swollen liver cells, and disorder of liver cell cords (Fig. S7C). Enlarged splenic nodules, unclear white pulp boundaries, and increased lymphocytes were observed in the spleen (Fig. S7D).

The pangolin *Erysipelothrix* sp. P66 strain is lethal to mice with an LD50 of 199 (Fig. 5B). Atrophy of liver sinuses, activation of Kupffer cells, and a small amount of neutrophil exudation were observed in the liver (Fig. S7E). Red blood cells were observed in the spleen (Fig. S7F). Partial rupture of alveolar walls, visible bleeding, and accompanying inflammatory cell exudation, primarily composed of lymphocytes and neutrophils, were observed in the lungs (Fig. S7G).

### Protective efficacy of commercial erysipelothrix vaccines against *Erysipelothrix* sp. P66

To evaluate the potential threat of the pangolin *Erysipelothrix* sp. P66 to the swine industry, we conducted a study to assess the protective effect of immunization with two commercial vaccines in mice challenged with the pangolin strain. Mice were vaccinated with GC42 and G4T10 (1 × 10^7^ CFU) and were then intradermally challenged with 2 × 10^4^ CFU of the pangolin strain after a period of 14 days. Following the challenge, all mice in the control group displayed clinical symptoms such as depression and anorexia, and succumbed within 3 days (Fig. 5C). Among the vaccinated mice, five out of eight died within 4–5 days, while only three mice survived in both vaccine groups (Fig. 5C). Consequently, neither of the commercial vaccines provided complete protection against the pangolin *Erysipelothrix* sp. P66 strain.

## DISCUSSION

We present evidence of the presence of *Corynebacterium ulcerans* and *Erysipelothrix* sp. bacteria in diseased Malaysian pangolins (*Manis javanica*) collected in 2019, designated as *C. ulcerans* P69 and *Erysipelothrix* sp. P66, respectively.

*C. ulcerans* can infect not only domestic animals such as goats and dogs but also a variety of wild animals including hedgehogs and monkeys (19–22). These findings suggest that both wildlife and domestic species have played roles in the transmission and evolution of this pathogen. Human infection with *C. ulcerans* can be fatal, and the frequency and severity of human infections appear to be increasing in many countries with some cases possibly originating from livestock (9, 23, 24). *C. ulcerans* strains were grouped into two lineages (25). *C. ulcerans* P69 clustered within the human isolate 4940 with 99.2% nucleotide identity across the whole genome (Fig. 2A; Fig. S2A). This raises the possibility that the pangolin *C. ulcerans* P69 has zoonotic potential. *C. ulcerans* P69 and *C. ulcerans* 4940 had 168 and 115 unique protein clusters, respectively. Of these, 21.4% (36/168) and 12.2% (14/115) were prophage genes (Fig. S3B and C), indicating that the presence of distinct prophages may be the major driving force behind the minor genomic differences between the two strains.

*C. ulcerans* is capable of producing diphtheria exotoxin and causing disease in domestic and wild animals. However, screening of prominent virulence factors revealed that the *C. ulcerans* P69 lacked the diphtheria toxin (*tox*) gene (Fig. 3A). Diphtheria toxin is widely used as a vaccine around the world to protect humans from the infection of corynebacteria. The lack of the *tox* gene suggests that the vaccine and diphtheria antitoxin may be of limited efficacy in this case (26). The analysis of virulence genes showed that this strain contained other known virulence-associated genes such as pld (phospholipase D) (Fig. 3A), which had 97.39% amino acid identity to human strain 809 associated with fatal pulmonary infection, and both strains exhibit similar LD50 (27). Strains that only produce phospholipase D but not diphtheria toxin are able to cause pneumonia and granulomatous nodules in the respiratory tract in humans (28). Hence, potential zoonotic pangolin *C. ulcerans* may pose a threat to public health.

According to the Clinical and Laboratory Standards Institute (CLSI) guidelines, *C. ulcerans* P69 exhibited multidrug resistance to five drugs, including penicillin and erythromycin, which are the first-line antibiotics recommended (Table 1). Notably, no resistance genes were detected in the genome of *C. ulcerans* P69. We speculate that this may be due to the presence of unknown resistance genes or an enhanced expression of drug efflux pumps (29, 30). Marosevic et al.’s study on the antibiotic resistance of 123 strains of *C. ulcerans* did not find penicillin resistance or multidrug-resistant strains (31). To our knowledge, *C. ulcerans* P69 is the first known strain of multidrug-resistant *C. ulcerans*, highlighting the need for ongoing investigation into antibiotic susceptibility in *C. ulcerans*.

*Erysipelothrix* sp. can infect a series of domestic animals, such as chickens, ducks, pigs, sheep, and dogs, and infections in humans principally occur as a result of contact with contaminated animals or their products (32, 33). *E. rhusiopathiae* is the pathogen of swine erysipelas, which has caused serious losses for the swine industry (34). It was thought that only *E. rhusiopathiae*, among the *Erysipelothrix* genus, had pathogenic significance. However, epidemiological investigations of pig and sheep farms where erysipelas outbreaks occurred revealed that in addition to the majority of *E. rhusiopathiae* isolated, other *Erysipelothrix* species were also found, which may play a more significant role in erysipelas outbreaks than previously believed (35, 36). In this study, the pangolin isolated *Erysipelothrix* sp. P66 strain was closely related to *Erysipelothrix* sp. strain 2-related strains isolated from humans, fish, and turkeys with a whole-genome identity of ~98.8% (Fig. 2B; Fig. S2B). The *Erysipelothrix* sp. strain 2-related isolates have been reported to be lethal to farmed fish (37), turkeys (38), as well as pigs (39). Human infection was also reported (40). *Erysipelothrix* sp. P66 exhibited similar LD50 values to highly virulent *E. rhusiopathiae* and *Erysipelothrix* sp. strain 2-related isolates (strain 715) (39). It may therefore pose a realistic threat to the health of pangolins and has a potential risk to other animals.

Pan-genome analysis of *Erysipelothrix* sp. strain 2-related isolates revealed that the functional categories of their differential genes belong to the “replication, recombination, and repair” group with the exception of unannotated genes (Fig. S4). This may reflect the robust genomic plasticity of *Erysipelothrix* sp. strain 2-related strains, which is advantageous for adaptation to diverse host environments (41). The surface protein spa located on bacterial cells is highly immunogenic (42). Previous studies have found that SpaC exists in all *Erysipelothrix* sp. strain 2-related isolates (43). In *Erysipelothrix* sp. P66, spaC is also present (Fig. 4A). The spaC immunoprotective domain of the isolates from pangolins (strain P66), pigs (strain 715), and turkeys (strain EsS2-6-Brazil and EsS2-7-Brazil) has high sequence identity with only a few amino acid substitutions (Fig. 4B). Previous studies have indicated that spaC can provide protective immunity against *E. rhusiopathiae* strains expressing heterologous spas, making it a potential candidate for vaccine for eradication of erysipelas (42, 44). Protection experiments with swine erysipelas attenuated vaccines showed that two commercially available attenuated vaccines (GC42, G4T10) carrying the spaA antigen protein had limited immune protective effect on mice challenged with *Erysipelothrix* sp. P66 (Fig. 5C). Therefore, the potential threat of this pathogen merits attention, and the impact of spaC protein on potential vaccine candidates warrants further investigation. Antimicrobial susceptibility testing suggests that *Erysipelothrix* sp. P66 showed resistance to vancomycin and gentamicin but was sensitive to penicillin and ceftiofur (Table 1). Thus, the treatment options for *Erysipelothrix* sp. P66 are similar to *Erysipelothrix rhusiopathiae*, which comprise penicillin and ceftiofur (45).

In this study we provide the first evidence that pangolins carry two bacteria: *C. ulcerans* and *Erysipelothrix* sp. Both bacteria can infect a series of wild and domesticated animals with potential zoonotic transmission to humans. Cases of human infection with *C. ulcerans* are increasing in many countries, while the infection case of *Erysipelothrix* sp. strain 2-related strain in humans has also been reported. As such, we cannot ignore the potential role of pangolins in the circulation of these two bacteria. Pangolins are also listed as critically endangered species on the IUCN Red List, and our research also provides a guide for the selection of antibiotics to treat bacterial infections in these critically endangered animals. Further research is needed to understand the diversity, prevalence, and virulence of bacterial pathogens in pangolins and their potential transmission or reverse transmission to humans and other animals.

## MATERIALS AND METHODS

### Bacterial isolation

Diseased Malaysian pangolins (*Manis javanica*) were collected in 2019. These pangolins were confiscated by the Customs and Department of Forestry of Guangdong Province, China, and brought into a rescue center. These samples were used for viral detections in our previous studies (2, 4, 46, 47). Sections of the lungs from pangolin P69 and the spleen from P66 were used to isolate bacteria using blood agar plates with 5% sheep blood. After incubation at 37°C for up to 48 hours, bacterial colonies were streaked onto blood agar plates and tryptic soy agar plates to obtain pure cultures. Both cultures were subjected to Gram staining and microscopic examination. Bacteriological species identification was performed by amplifying the 16S rRNA using the primer pair (8F: AGAGTTTGATCCTGGCTCAG; U1492R: GGTTACCTTGTTACGACTT) (48).

### Animal infection experiments

Eight-week-old male KM mice were obtained from Southern Medical University Animal Center (Guangzhou, China). To assess the virulence of *C. ulcerans* and *Erysipelothrix* sp., 30 KM mice were randomly divided into five infection groups and one control group for each bacterium. For *C. ulcerans*, mice were inoculated intraperitoneally (i.p.) with 0.2 mL bacterial suspensions with (3.5 × 10^9^ to 3.5 × 10^5^ CFU/mL), while in the case of *Erysipelothrix* sp., mice were inoculated i.p. with 0.2 mL bacterial suspensions (3.7 × 10^4^ to 3.7 CFU/mL). The control group was injected with 200 µL of 0.9% physiological saline, and mortality was recorded for 14 days. The LD50 value was estimated using the method described by Reed and Muench (1938).

### Vaccination and bacterial challenge of the pangolin *Erysipelothrix* sp. in mice

To investigate the protective effect of two swine erysipelas attenuated vaccines (GC42 and G4T10) against challenge with the pangolin *Erysipelothrix* sp. strain, 32 KM mice were randomly divided into two vaccinated groups (eight mice per group) and one control group (six mice). The mice in the vaccinated groups were intraperitoneally vaccinated with 0.2 mL of GC42 and G4T10 (1 × 10^7^ CFU/mL), respectively, while the control group was inoculated with 0.9% physiological saline. After 14 days of vaccination, all three groups of mice were challenged with 100 LD50 of *Erysipelothrix* sp. The clinical symptoms, morbidity, and mortality were observed and recorded for 14 days. Necropsy was performed on deceased mice to isolate and identify the bacteria.

### Histopathological examination

To ensure optimal tissue fixation for histopathological examination of bacterial-infected tissue, tissues from mice were placed in a 4% paraformaldehyde solution for 24 hours. Subsequently, the tissue blocks were sequentially sectioned at a thickness of approximately 3–4 μm using a microtome, and the resulting sections were carefully mounted on clean glass slides. To facilitate staining, paraffin was removed, and the sections were rehydrated using graded ethanol solutions and distilled water. The sections were then successfully stained using the Hematoxylin-Eosin Staining Kit (Servicebio, China).

### Antimicrobial susceptibility testing

Antimicrobial susceptibility testing profiles of the two strains were determined using the broth dilution method recommended by CLSI with *E. coli* ATCC 25922 and *Staphylococcus aureus* ATCC 29213 as the quality control strains. Susceptibility to 13 antibiotics—erythromycin (ERY), tilmicosin (CLD), oxacillin (OXA), penicillin G (TIL), vancomycin (VAN), clindamycin (SXT), gentamicin (TET), cefoxitin (PEN), cotrimoxazole (GEN), tetracycline (FOX), ceftiofur (CEF), ciprofloxacin (CIP), and florfenicol (FFC)—was tested, and the results were partly interpreted according to CLSI documents.

### Bacterial genome sequencing and bioinformatic analysis

DNA of the two bacterial isolates was extracted with SDS method (49). Sequencing libraries were generated using NEBNext Ultra DNA Library Prep Kit for Illumina (NEB, USA) following the manufacturer’s protocol. They were sequenced by combining Oxford Nanopore platform and Illumina NovaSeq PE150 Unicycler (v0.4.7) (50), such that their genomes were assembled using a combination of short-read and long-read sequences. A circular genome map was plotted using Proksee (51). The genome sequences of 56 published *Corynebacterium* and *Erysipelothrix* strains were obtained from the NCBI Reference Sequence Database for comparative analyses (Table S1). Whole-genome ANI was calculated using fastANI with arguments “-fragment length 1000” (52). Roary was used to calculate the pan-genome and identify the core genes (53). Maximum likelihood (ML) phylogenetic trees were estimated using IQ-TREE with 1,000 bootstrap replicates (54) and visualized in FigTree (http://tree.bio.ed.ac.uk/software/figtree/). The core genes of 43 *C*. *ulcerans* strains were used for estimating ML phylogenetic trees and comparative genomic analysis (Table S2). Similarly, the core genes of 15 *Erysipelothrix* spp. strains (Table S3) and four strains related to *Erysipelothrix* sp. strain 2 (Table S4) were utilized for estimating ML phylogenetic trees and comparative genomic analysis, respectively.

All genome sequences were annotated by Prokka (v1.14.6) (55). Protein category (COG) analysis was performed using the eggNOG-mapper (56). The ST type of *C. ulcerans* strains was determined using MLST (https://github.com/tseemann/mlst) (57). Virulence and antimicrobial resistance genes were identified using the VFDB (58) and CARD (59) databases, employing BLAST searches (identity > 70%, coverage > 90%) (60). For *Erysipelothrix* sp. P66, an additional search was conducted for 13 genes (*spa*, *nanH.1*, *nanH.2*, *cpsA*, ERH_1356, *intl-like*, *rspA*, *rspB*, *algI*, *sub*, *hlyA*, *fbpA*, and *hlyIII*) related to the virulence traits of *E. rhusiopathiae* (61). Phastest (62) was employed for prophage identification. Connections between prophage CDSs were identified using Blastp (identity > 80%; subject cover > 60%). Pairs with more than 10 similar CDSs were then filtered for constructing the network diagram. pyGenomeViz (https://github.com/moshi4/pyGenomeViz) was utilized to visualize the genomic features and sequence similarity comparisons among multiple prophages. The amino acid sequence alignment of the *spaC* protein was conducted using MAFFT with identity values visualized using Geneious (63). Heatmaps, bar graphs, and Venn diagrams were generated using the R package through Hiplot (https://hiplot.com.cn/), while the survival curve analysis was performed on the cloud platform OmicStudio (64).

## Data Availability

Sequence reads generated in this study are available from the NCBI Sequence Read Archive (SRA) database under BioProject accession number PRJNA1102293.

## SUPPLEMENTAL MATERIAL

Figure S1 msphere.00551-24-s0001.tif Gram staining of *C. ulcerans* P69 and *Erysipelothrix* sp. strain P66.

Figure S2 msphere.00551-24-s0002.tif Average nucleotide identity matrix based on alignments of whole assembled genomes.

Figure S3 msphere.00551-24-s0003.tif Core genome functional categories and unique protein clusters in 43 *C. ulcerans* strains.

Figure S4 msphere.00551-24-s0004.tif Core genome functional categories and unique protein clusters in *Erysipelothrix* sp. strain 2.

Figure S5 msphere.00551-24-s0005.tif Comparative genomic analysis of prophage genomes of *C. ulcerans*.

Figure S6 msphere.00551-24-s0006.tif Amino acid sequence similarities among Spas from 23 *Erysipelothrix* strains.

Figure S7 msphere.00551-24-s0007.tif The body weight changes and pathological changes in mice infected with bacteria.

Supplemental Material msphere.00551-24-s0008.docx Supplemental figure legends and tables.

ASM does not own the copyrights to Supplemental Material that may be linked to, or accessed through, an article. The authors have granted ASM a non-exclusive, world-wide license to publish the Supplemental Material files. Please contact the corresponding author directly for reuse.
