# Supplementary figures and images for: Identification of Corynebacterium ulcerans and Erysipelothrix sp. in Malayan pangolins—a potential threat to public health?
Source: mSphere. 2024 Sep 30;9(10):e00551-24. doi: 10.1128/msphere.00551-24 (PMC11520285; doi:10.1128/msphere.00551-24)

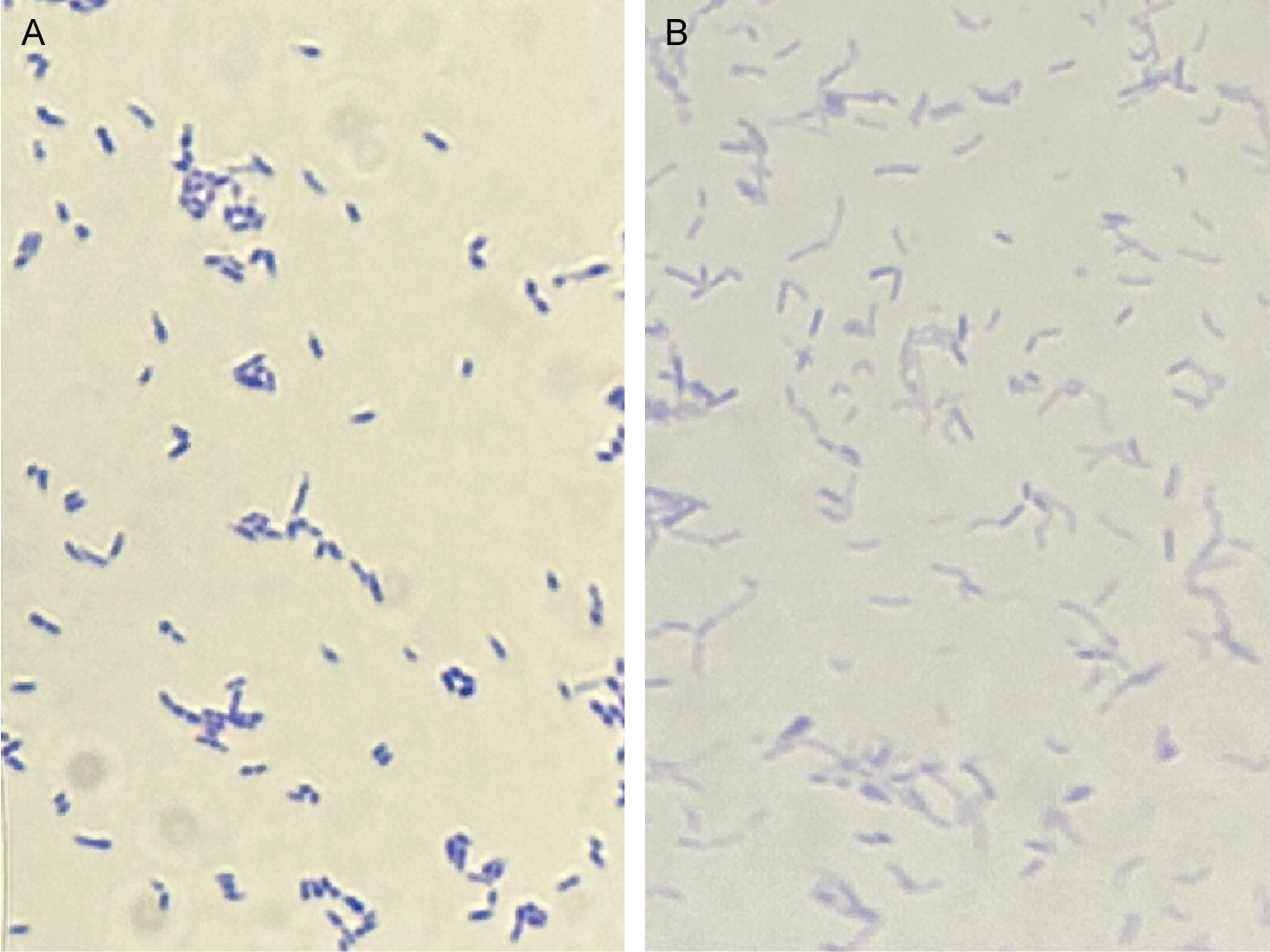

Supplement: Figure S1 — Gram staining of C. ulcerans P69 and Erysipelothrix sp. strain P66. [file msphere.00551-24-s0001.tif]

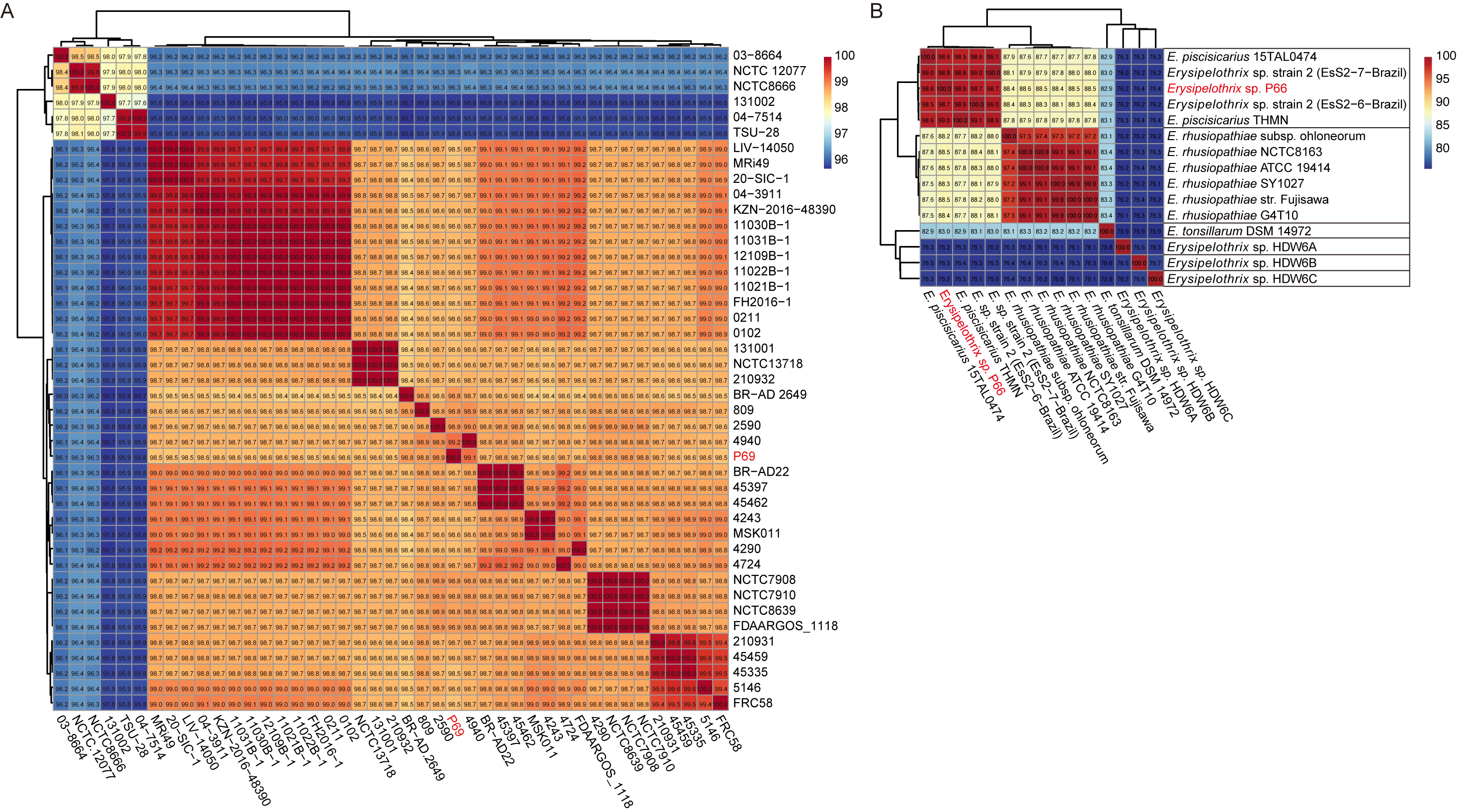

Supplement: Figure S2 — Average nucleotide identity matrix based on alignments of whole assembled genomes. [file msphere.00551-24-s0002.tif]

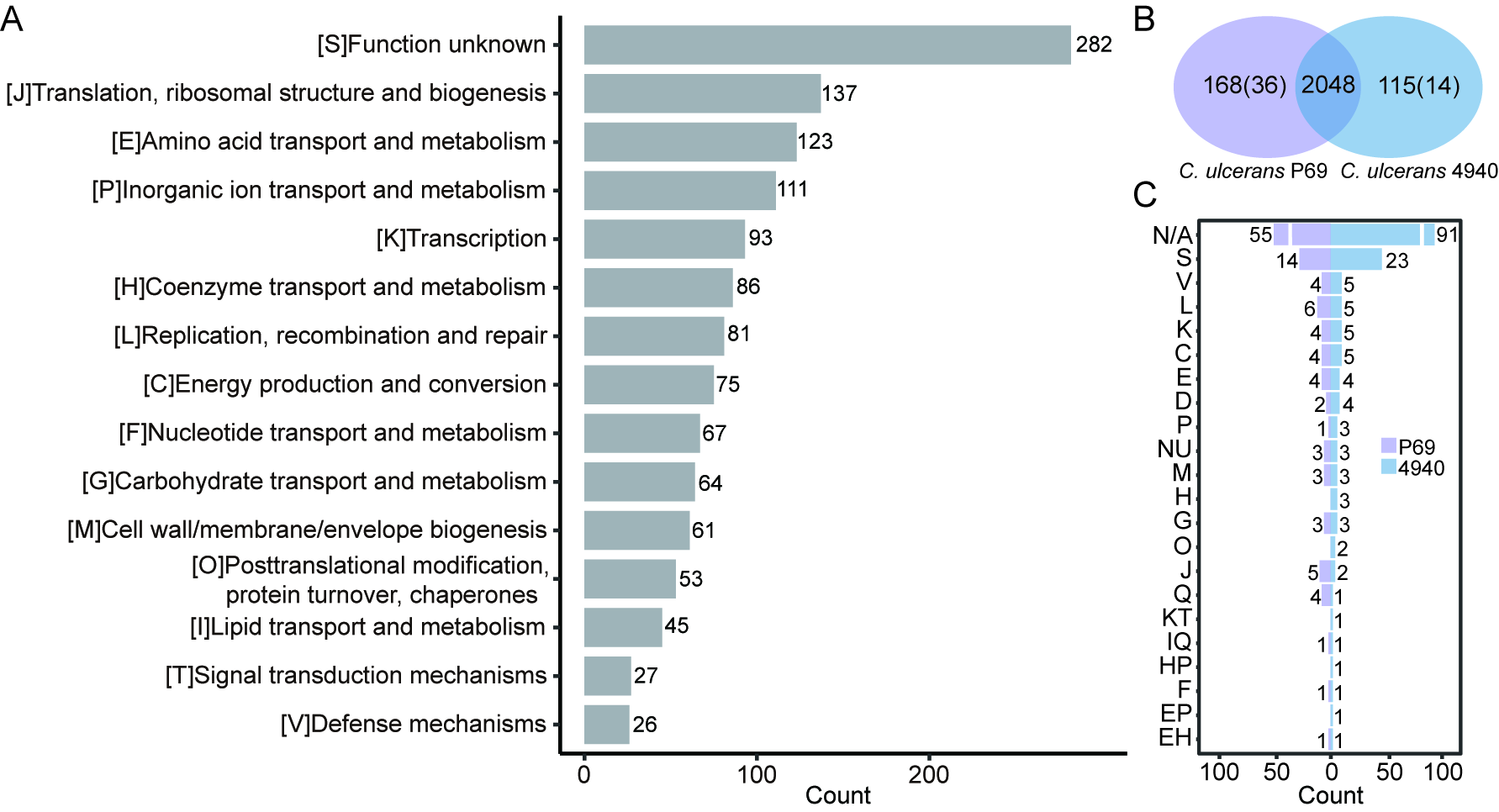

Supplement: Figure S3 — Core genome functional categories and unique protein clusters in 43 C. ulcerans strains. [file msphere.00551-24-s0003.tif]

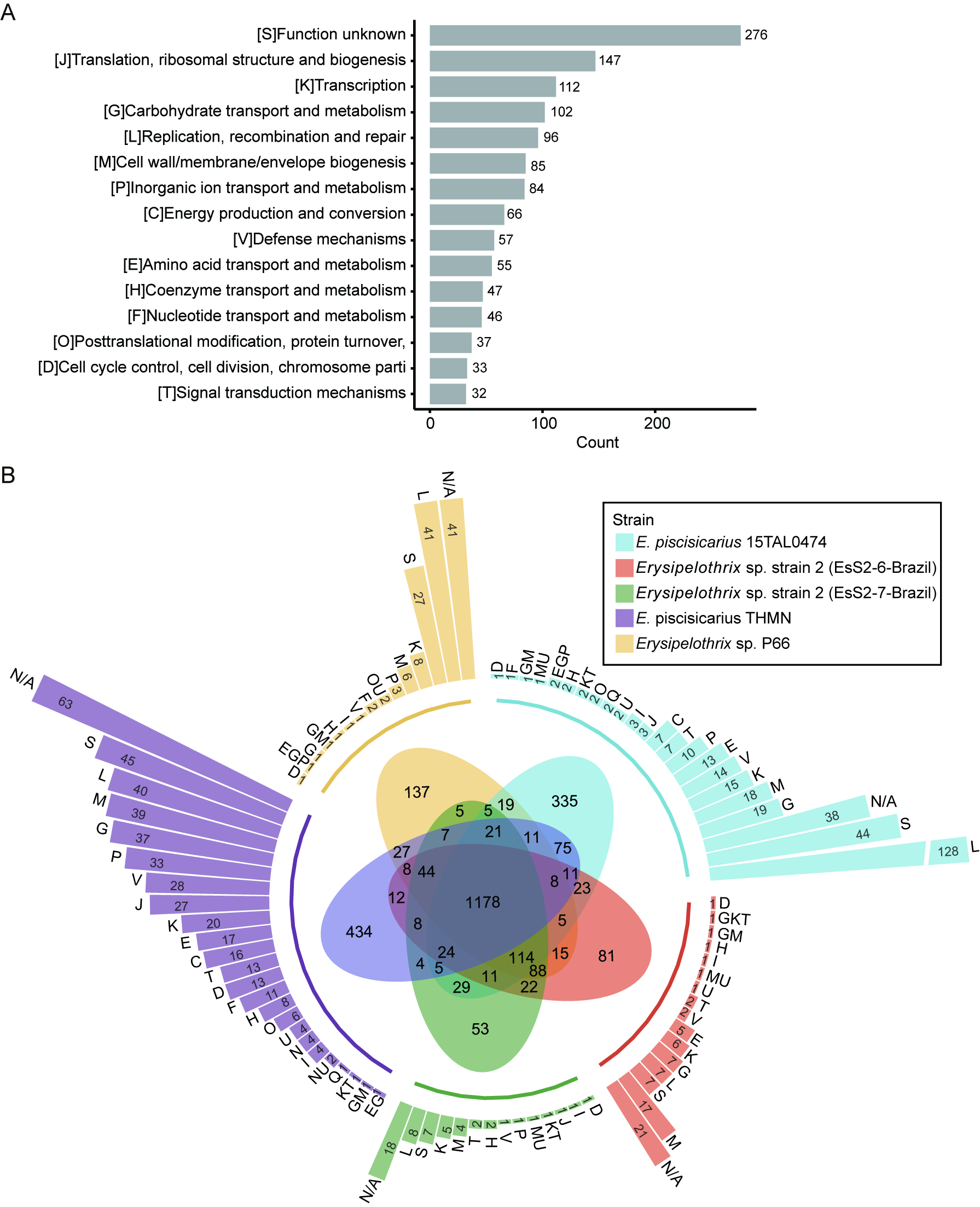

Supplement: Figure S4 — Core genome functional categories and unique protein clusters in Erysipelothrix sp. strain 2. [file msphere.00551-24-s0004.tif]

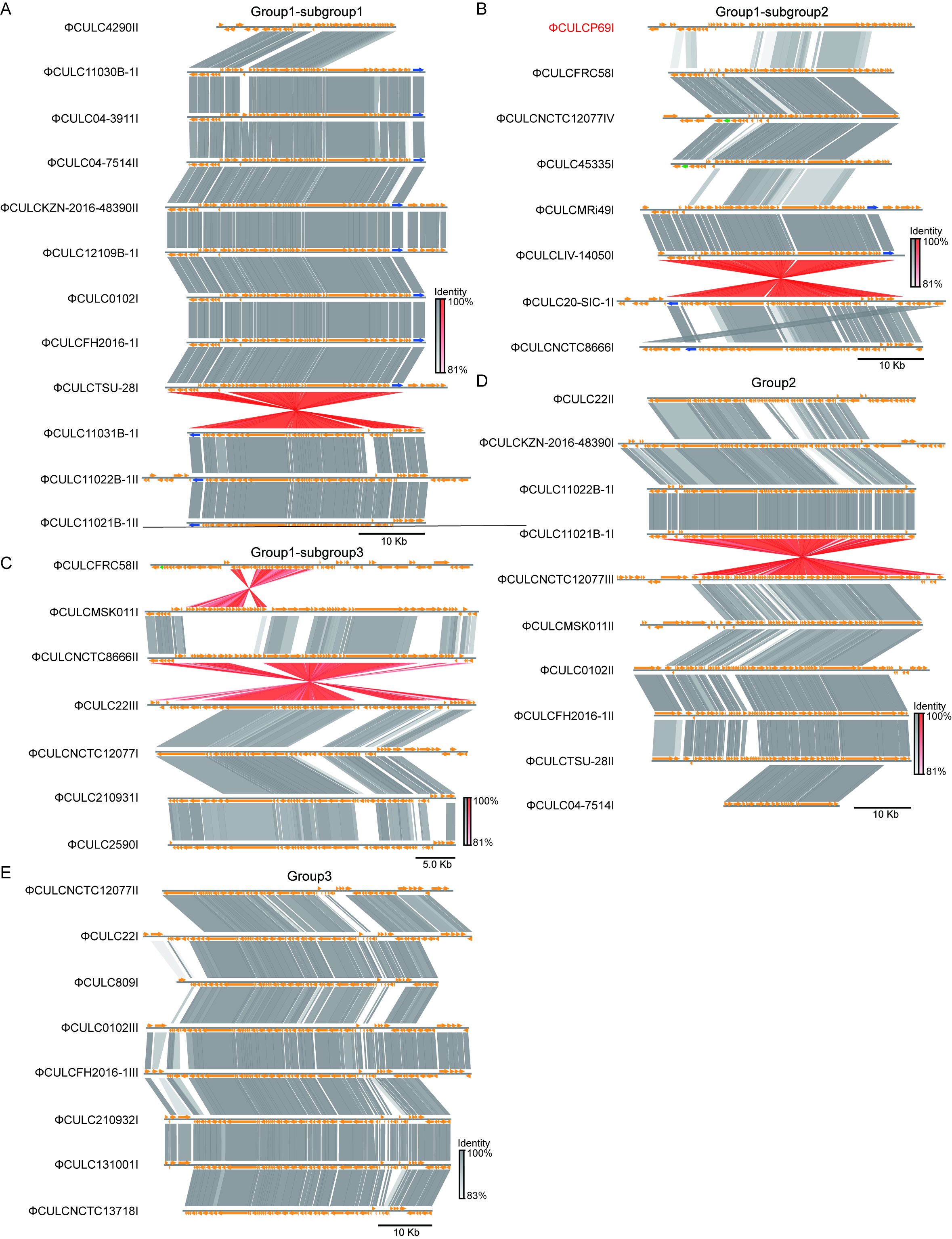

Supplement: Figure S5 — Comparative genomic analysis of prophage genomes of C. ulcerans. [file msphere.00551-24-s0005.tif]

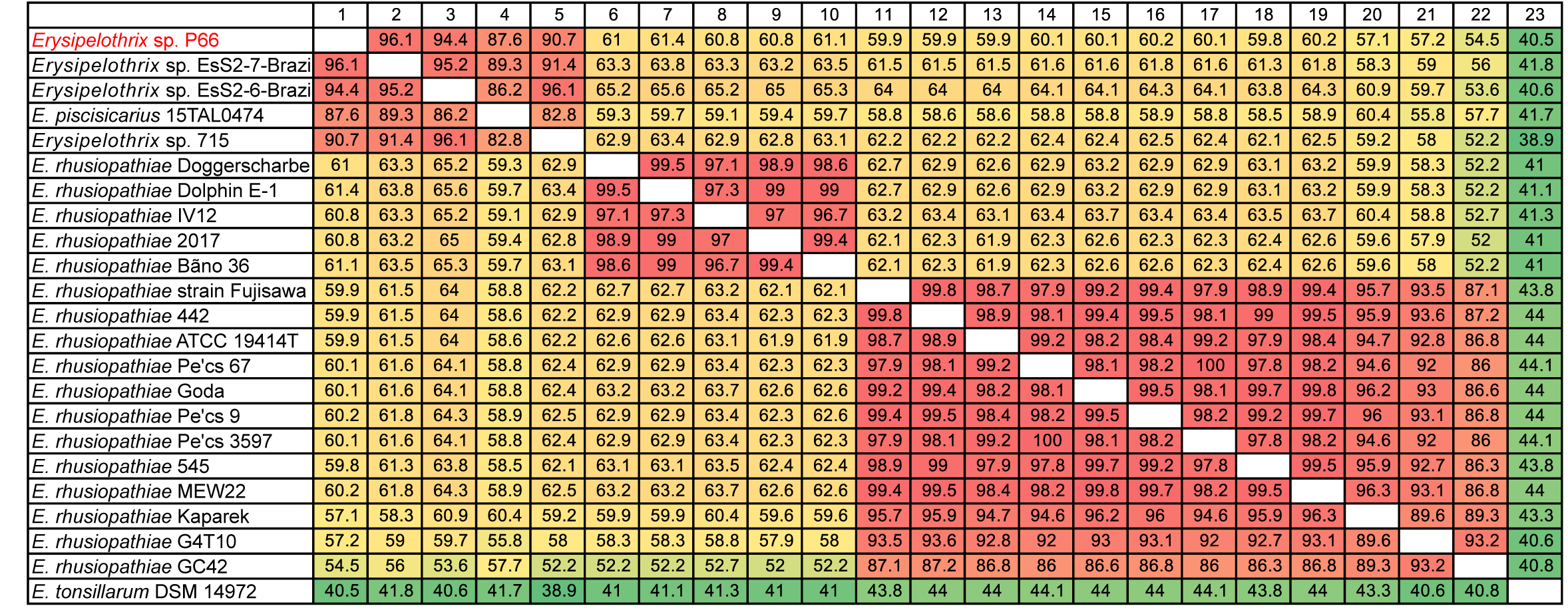

Supplement: Figure S6 — Amino acid sequence similarities among Spas from 23 Erysipelothrix strains. [file msphere.00551-24-s0006.tif]

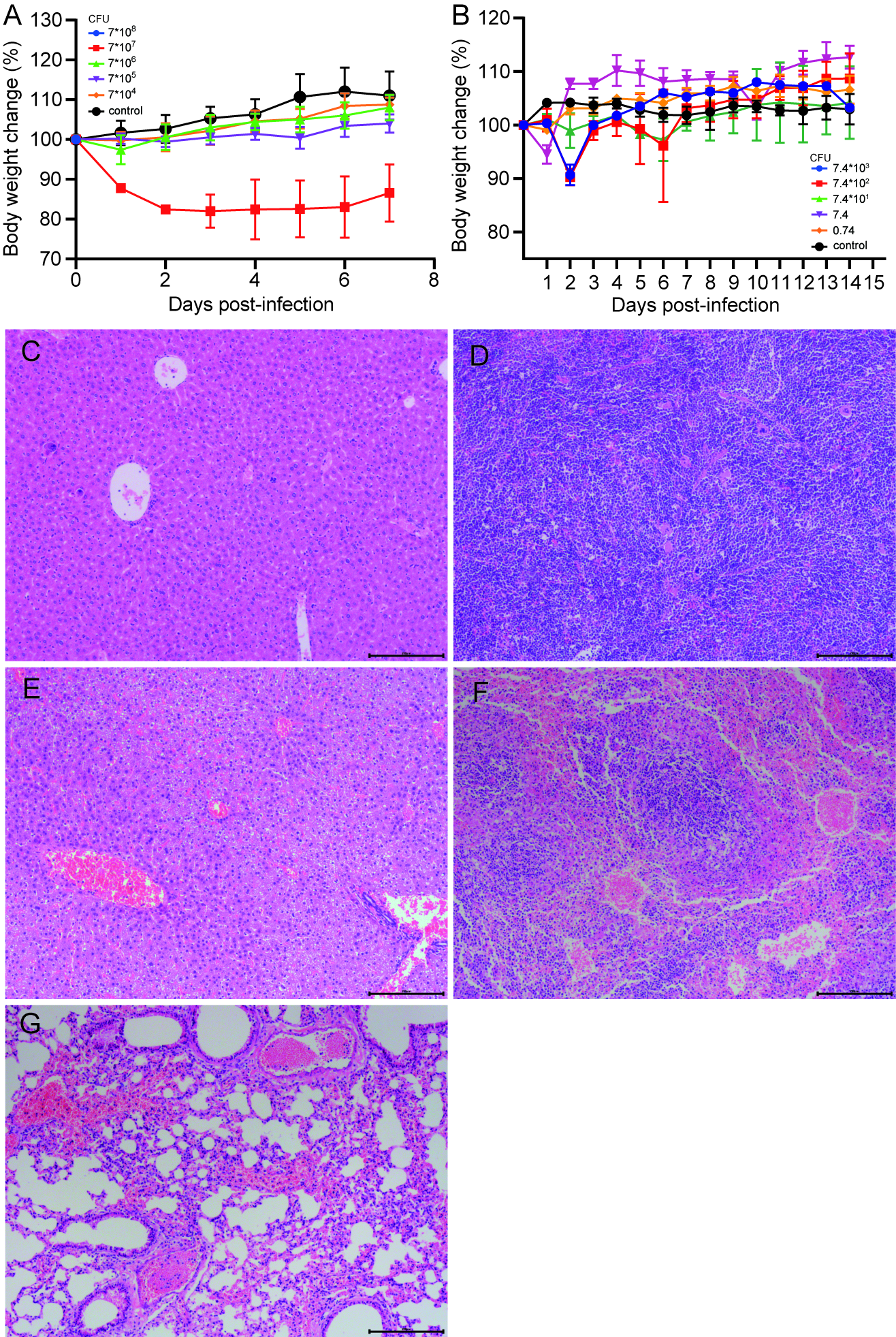

Supplement: Figure S7 — The body weight changes and pathological changes in mice infected with bacteria. [file msphere.00551-24-s0007.tif]
