# Supplementary material for: Identification of Corynebacterium ulcerans and Erysipelothrix sp. in Malayan pangolins—a potential threat to public health?
Source: mSphere. 2024 Sep 30;9(10):e00551-24. doi: 10.1128/msphere.00551-24 (PMC11520285; doi:10.1128/msphere.00551-24)
Supplement: Supplemental Material — Supplemental figure legends and tables. [file msphere.00551-24-s0008.docx]

**Supplementary Figure 1. Gram staining (oil immersion, ×1,000).** *C. ulcerans* P69 (A) and *Erysipelothrix* sp. P66 (B).

**Supplementary Figure 2. Average nucleotide identity matrix based on alignments of whole assembled genomes.** (A) Average nucleotide identity of 43 *Corynebacterium* isolates. (B) Average nucleotide identity of 15 *Erysipelothrix* isolates.

**Supplementary Figure 3. Core genome functional categories and unique protein clusters in 43 *C. ulcerans* strains.** (A) Distribution of core genes across functional categories based on Clusters of Orthologous Groups (COG) classification. The top 15 functional categories are shown, with the number of genes in each category indicated. (B) Venn diagram illustrating the number of shared and unique protein clusters between *C. ulcerans* P69 and 4940. (C) Histogram displaying the number of unique protein clusters in each COG functional category for strains P69 and 4940.

**Supplementary Figure 4. Core genome functional categories and unique protein clusters in *Erysipelothrix* sp. Strain 2 (isolates 15TAL0474, THMN, EsS2-6-Brazil, EsS2-7-Brazil).** (A) Distribution of core genes across functional categories based on Clusters of Orthologous Groups (COG) classification. The top 15 functional categories are shown, with the number of genes in each category indicated. (B) Venn diagram in the center illustrating the number of shared and unique protein clusters between four strains related to *Erysipelothrix* sp. strain 2. Histogram in the outer circle displaying the number of unique protein clusters in each COG functional category for four strains related to *Erysipelothrix* sp. strain 2.

**Supplementary Figure 5. Comparative genomic analysis of prophage genomes of *C. ulcerans*.** Genomic comparisons were visualized using pyGenomeViz, with regions of high amino acid identity between coding sequences (CDSs) indicated by shaded areas of varying intensities. Forward connections are shown in gray, while reverse connections are highlighted in red. The tox virulence gene CDSs are marked in blue, while the RhuM virulence gene CDSs are marked in green. Grouped according to the information in Figure 3B: (A) group1-subgroup1, (B) group1-subgroup2, (C) group1-subgroup3, (D) group2, and (E) group3.

**Supplementary Figure 6. Amino acid sequence similarities among Spas from 23 *Erysipelothrix* strains.**

**Supplementary Figure 7. The body weight changes and pathological changes in mice infected with bacteria.** The body weight changes of mice infected with different doses of bacteria (A) *C. ulcerans* P69 at 7*10^8^, 7*10^7^, 7*10^6^, 7*10^5^ and 7*10^4^ CFU. (B) *Erysipelothrix* sp. P66 at 7.4*10^3^, 7.4*10^2^, 7.4*10^1^, 7.4 and 0.74 CFU. Pathological changes of the liver (C) and spleen (D) tissues from mice inoculated with *C. ulcerans* P69. Pathological changes of the liver (E), spleen (F), and lung (G) tissues from mice inoculated with *Erysipelothrix* sp. P66. The tissues were stained with hematoxylin and eosin for histological evaluation. The images were magnified at a 100x magnification.

**Supplementary Table S1. The reference genomes utilized in this study.**

| Strain | Length | Date | Assembly Level | Assembly Accession Number |
| --- | --- | --- | --- | --- |
| *C. ulcerans* 04-3911 | 2492680 | 2015 | Contig | GCF_001298275.1 |
| *C. ulcerans* KZN-2016-48390 | 2541110 | 2016 | Contig | GCF_001876325.1 |
| *C. ulcerans* 4724 | 2470980 | 2020 | Contig | GCF_016019795.1 |
| *C. ulcerans* FRC58 | 2542597 | 2015 | Complete Genome | GCF_000499805.2 |
| *C. ulcerans* 210932 | 2484335 | 2014 | Complete Genome | GCF_000767415.1 |
| *C. ulcerans* 131001 | 2483321 | 2015 | Complete Genome | GCF_001281445.1 |
| *C. ulcerans* NCTC13718 | 2481768 | 2018 | Complete Genome | GCF_900475605.1 |
| *C. ulcerans* 5146 | 2466435 | 2014 | Complete Genome | GCF_000769635.1 |
| *C. ulcerans* 210931 | 2509428 | 2014 | Complete Genome | GCF_000767645.1 |
| *C. ulcerans* 45335 | 2518913 | 2020 | Contig | GCF_013135495.1 |
| *C. ulcerans* NCTC8666 | 2542414 | 2018 | Contig | GCF_900447245.1 |
| *C. ulcerans* NCTC 12077 | 2616289 | 2013 | Contig | GCF_000498915.1 |
| *C. ulcerans* FH2016-1 | 2579134 | 2019 | Complete Genome | GCF_009002285.1 |
| *C. ulcerans* 0211 | 2579078 | 2019 | Complete Genome | GCF_008995395.1 |
| *C. ulcerans* 0102 | 2579188 | 2012 | Complete Genome | GCF_000306825.1 |
| *C. ulcerans* 12109B-1 | 2472441 | 2022 | Contig | GCF_024341765.1 |
| *C. ulcerans* 11031B-1 | 2471864 | 2022 | Scaffold | GCF_024341785.1 |
| *C. ulcerans* 11021B-1 | 2520008 | 2022 | Scaffold | GCF_024341845.1 |
| *C. ulcerans* 11022B-1 | 2520022 | 2022 | Contig | GCF_024341825.1 |
| *C. ulcerans* 11030B-1 | 2471752 | 2022 | Scaffold | GCF_024341805.1 |
| *C. ulcerans* 809 | 2502095 | 2011 | Complete Genome | GCF_000215645.1 |
| *C. ulcerans* BR-AD22 | 2606374 | 2011 | Complete Genome | GCF_000215665.1 |
| *C. ulcerans* 45397 | 2446713 | 2020 | Contig | GCF_013135455.1 |
| *C. ulcerans* 45462 | 2446966 | 2020 | Contig | GCF_013135435.1 |
| *C. ulcerans* 03-8664 | 2428683 | 2016 | Contig | GCF_001298285.2 |
| *C. ulcerans* TSU-28 | 2522327 | 2023 | Complete Genome | GCF_030295465.1 |
| *C. ulcerans* 04-7514 | 2497845 | 2015 | Contig | GCF_001302345.1 |
| *C. ulcerans* NCTC7910 | 2453761 | 2017 | Complete Genome | GCF_900187135.1 |
| *C. ulcerans* FDAARGOS_1118 | 2453751 | 2021 | Complete Genome | GCF_016727365.1 |
| *C. ulcerans* NCTC8639 | 2453749 | 2018 | Complete Genome | GCF_900475775.1 |
| *C. ulcerans* 4940 | 2419371 | 2018 | Contig | GCF_002872395.1 |
| *C. ulcerans* MRi49 | 2527244 | 2019 | Complete Genome | GCF_009789155.1 |
| *C. ulcerans* LIV-14050 | 2513055 | 2020 | Complete Genome | GCF_014792805.1 |
| *C. ulcerans* 20-SIC-1 | 2510020 | 2021 | Contig | GCF_016765755.1 |
| *C. ulcerans* NCTC7908 | 2453674 | 2018 | Complete Genome | GCF_900475635.1 |
| *C. ulcerans* 45459 | 2516702 | 2020 | Contig | GCF_013135535.1 |
| *C. ulcerans* 4290 | 2604730 | 2020 | Contig | GCF_016019835.1 |
| *C. ulcerans* MSK011 | 2533710 | 2023 | Scaffold | GCF_030232465.1 |
| *C. ulcerans* 4243 | 2512771 | 2020 | Contig | GCF_016019855.1 |
| *C. ulcerans* BR-AD 2649 | 2541476 | 2018 | Contig | GCF_002866485.1 |
| *C. ulcerans* 2590 | 2501366 | 2018 | Contig | GCF_002866525.1 |
| *C. ulcerans* 131002 | 2434569 | 2015 | Complete Genome | GCF_000968945.1 |
| *E. piscisicarius* 15TAL0474 | 1722938 | 2018 | Complete Genome | GCF_003931795.1 |
| *E. piscisicarius* THMN | 1470447 | 2024 | Chromosome | GCF_036549255.1 |
| *E. rhusiopathiae* NCTC8163 | 1770411 | 2018 | Complete Genome | GCF_900637845.1 |
| *E. rhusiopathiae* G4T10 | 1770505 | 2019 | Complete Genome | GCF_006384935.1 |
| *E. rhusiopathiae* ATCC 19414 | 1746468 | 2011 | Contig | GCF_000160815.2 |
| *E. rhusiopathiae* str. Fujisawa | 1787941 | 2011 | Complete Genome | GCF_000270085.1 |
| *E. rhusiopathiae* subsp. ohloneorum | 1782830 | 2023 | Complete Genome | GCF_035066585.1 |
| *E. rhusiopathiae* SY1027 | 1752910 | 2013 | Complete Genome | GCF_000404205.1 |
| *E.* sp. HDW6A | 2043172 | 2020 | Complete Genome | GCF_011400295.1 |
| *E.* sp. HDW6B | 2255181 | 2020 | Complete Genome | GCF_011301115.1 |
| *E.* sp. HDW6C | 2357642 | 2020 | Complete Genome | GCF_011299615.1 |
| *E.* sp. strain 2 (EsS2-6-Brazil) | 1739138 | 2021 | Scaffold | GCF_016617625.1 |
| *E.* sp. strain 2 (EsS2-7-Brazil) | 1714240 | 2021 | Scaffold | GCF_016617655.1 |
| *E. tonsillarum* DSM 14972 | 1931022 | 2013 | Scaffold | GCF_000373785.1 |

**Supplementary Table S2. Pan-genome analysis of 43 *C. ulcerans* strains.**

|  | Annotation | Number of genes |
| --- | --- | --- |
| Core genes | (99% <= strains <= 100%) | 1601 |
| Soft core genes | (95% <= strains < 99%) | 174 |
| Shell genes | (15% <= strains < 95%) | 779 |
| Cloud genes | (0% <= strains < 15%) | 1878 |
| Total genes | (0% <= strains <= 100%) | 4432 |

**Supplementary Table S3. Pan-genome analysis of 15 *Erysipelothrix* sp. strains.**

|  | Annotation | Number of genes |
| --- | --- | --- |
| Core genes | (99% <= strains <= 100%) | 10 |
| Soft core genes | (95% <= strains < 99%) | 0 |
| Shell genes | (15% <= strains < 95%) | 2490 |
| Cloud genes | (0% <= strains < 15%) | 9180 |
| Total genes | (0% <= strains <= 100%) | 11680 |

**Supplementary Table S4. Pan-genome analysis of *Erysipelothrix* sp. P66 and the four strains related to *E.* sp. strain 2 (isolates 15TAL0474, THMN, EsS2-6-Brazil, EsS2-7-Brazil).**

|  | Annotation | Number of genes |
| --- | --- | --- |
| Core genes | (99% <= strains <= 100%) | 1178 |
| Soft core genes | (95% <= strains < 99%) | 0 |
| Shell genes | (15% <= strains < 95%) | 1641 |
| Cloud genes | (0% <= strains < 15%) | 0 |
| Total genes | (0% <= strains <= 100%) | 2819 |

**Supplementary Table S5. Prophage regions identified in *C. ulcerans* strains.**

| Strain | Prophage identifier | region position (bp) | region length | GC content |
| --- | --- | --- | --- | --- |
| P69 | I | 1126721-1167090 | 40.3Kb | 51.43% |
| NCTC 12077 | I | 9949-49282 | 39.3Kb | 55.72% |
| NCTC 12077 | II | 2338-55981 | 53.6Kb | 53.07% |
| NCTC 12077 | III | 172936-230844 | 57.9Kb | 55.46% |
| NCTC 12077 | IV | 393480-429250 | 35.7Kb | 52.67% |
| 210931 | I | 2011703-2047951 | 36.2Kb | 55.36% |
| 04-3911 | I | 133116-168977 | 35.8Kb | 52.70% |
| 04-7514 | I | 2648-22792 | 20.1Kb | 56.65% |
| 04-7514 | II | 39342-75446 | 36.1Kb | 52.73% |
| 2590 | I | 65430-101644 | 36.2Kb | 55.38% |
| 45335 | I | 17060-50423 | 33.3Kb | 52.18% |
| 4290 | I | 287758-314757 | 27Kb | 53.52% |
| 11031B-1 | I | 510300-546142 | 35.8Kb | 52.56% |
| 11030B-1 | I | 298432-334312 | 35.8Kb | 52.57% |
| 11022B-1 | I | 197078-244178 | 47.1Kb | 55.64% |
| 11022B-1 | II | 543531-593441 | 49.9Kb | 53.40% |
| 11021B-1 | I | 197019-244119 | 47.1Kb | 55.64% |
| 11021B-1 | II | 557442-593489 | 36Kb | 52.76% |
| MSK011 | I | 314899-357130 | 42.2Kb | 55.28% |
| MSK011 | II | 244979-294421 | 49.4Kb | 54.25% |
| NCTC8666 | I | 1215779-1258664 | 42.8Kb | 52.32% |
| NCTC8666 | II | 1863299-1904637 | 41.3Kb | 55.38% |
| BR-AD22 | I | 1277188-1338138 | 60.9Kb | 52.82% |
| BR-AD22 | II | 1855855-1902844 | 46.9Kb | 55.68% |
| BR-AD22 | III | 2115662-2157034 | 41.3Kb | 54.96% |
| 809 | I | 1276578-1324808 | 48.2Kb | 53.32% |
| 0102 | I | 177515-213562 | 36Kb | 52.75% |
| 0102 | II | 515223-567078 | 51.8Kb | 55.44% |
| 0102 | III | 1357320-1416800 | 59.4Kb | 52.84% |
| FH2016-1 | I | 176903-212950 | 36Kb | 52.76% |
| FH2016-1 | II | 514612-559226 | 44.6Kb | 55.58% |
| FH2016-1 | III | 1356506-1416919 | 60.4Kb | 52.92% |
| TSU-28 | I | 167082-209702 | 42.6Kb | 53.02% |
| TSU-28 | II | 500786-546246 | 45.4Kb | 55.46% |
| 210932 | I | 1258238-1311114 | 52.8Kb | 52.87% |
| 131001 | I | 1257974-1310806 | 52.8Kb | 52.86% |
| FRC58 | I | 302275-336368 | 34Kb | 51.67% |
| FRC58 | II | 1783307-1823902 | 40.5Kb | 53.89% |
| MRi49 | I | 171829-214506 | 42.6Kb | 52.53% |
| LIV-14050 | I | 2469058-2506376 | 37.3Kb | 52.24% |
| NCTC13718 | I | 1821256-1867235 | 45.9Kb | 53.45% |
| KZN-2016-48390 | I | 176414-233777 | 57.3Kb | 55.59% |
| KZN-2016-48390 | II | 293396-335968 | 42.5Kb | 53.01% |
| 20-SIC-1 | I | 89830-139833 | 50Kb | 52.99% |
| 12109B-1 | I | 292583-335158 | 42.5Kb | 53.01% |

**Supplementary Table S6. The results of the Blastp analysis of potential genes associated with virulence in *Erysipelothrix* sp. P66 strains.**

| Query | Gene/Locus Tag | Gene Product/Predicted Function | Identity | Coverage |
| --- | --- | --- | --- | --- |
| FKIMDJLJ_00418 | *algI*; ERH_0402 | Alginate-O-acetyltransferase/resistance to phagocytosis | 86.2 | 100 |
| FKIMDJLJ_00167 | *cpsA*; ERH_0157 | Capsule polysaccharide synthesis gene (glycosyl transferase)/resistance to complement | 89.9 | 100 |
| FKIMDJLJ_01400 | ERH_1356 | ABC transporter metal-binding lipoprotein/adhesion of host cells | 92.9 | 100 |
| FKIMDJLJ_01069 | *fbpA*; ERH_1034 | Fibronectin-binding protein/adhesion | 90.4 | 100 |
| FKIMDJLJ_00670 | *hlyIII*; ERH_0649 | Hyaluronidase/adhesion-promoting factor | 94.1 | 100 |
| FKIMDJLJ_00156 | *hylA*; ERH_0150 | Haemolysin/lytic activity on red blood cells | 84.6 | 100 |
| FKIMDJLJ_01526 | *intl*; ERH_1472 | Internalin/invasion of epithelial cells | 78.3 | 97.6 |
| FKIMDJLJ_00307 | *nanH.1*; ERH_0299 | Neuraminidase/spreading factor | 94 | 100 |
| FKIMDJLJ_00803 | *nanH.2*; ERH_0761 | Neuraminidase/spreading factor | 75.4 | 100 |
| FKIMDJLJ_00689 | *rspA*; ERH_0668 | Rhusiopathiae surface protein/biofilm formation | 96.5 | 100 |
| FKIMDJLJ_00690 | *rspB*; ERH_0669 | Rhusiopathiae surface protein/biofilm formation | 94.9 | 100 |
| FKIMDJLJ_00277 | *sub*; ERH_0260 | Cell-envelope associated proteinase, subtilase family | 88.3 | 100 |
| FKIMDJLJ_00103 | *spaC*; AB238210 | Surface protection antigen C | 90.8 | 100 |
